# Genotyping of *Francisella tularensis* subsp. *holarctica* from Hares in Germany

**DOI:** 10.3390/microorganisms8121932

**Published:** 2020-12-05

**Authors:** Jörg Linde, Timo Homeier-Bachmann, Alexandra Dangel, Julia M. Riehm, David Sundell, Caroline Öhrman, Mats Forsman, Herbert Tomaso

**Affiliations:** 1 Institute of Bacterial Infections and Zoonoses, Friedrich-Loeffler-Institut, 07743 Jena, Germany; herbert.tomaso@fli.de; 2 Institute of Epidemiology, Friedrich-Loeffler-Institut, 17493 Greifswald-Insel Riems, Germany; Timo.Homeier@fli.de; 3 Bayerisches Landesamt für Gesundheit und Lebensmittelsicherheit, 85764 Oberschleißheim, Germany; Alexandra.dangel@lgl.bayern.de (A.D.); Julia.Riehm@lgl.bayern.de (J.M.R.); 4 CBRN Defence and Security, Swedish Defence Research Agency (FOI), SE-901 82 Umeå, Sweden; david.sundell@foi.se (D.S.); caroline.ohrman@foi.se (C.Ö.); mats.forsman@foi.se (M.F.)

**Keywords:** *Francisella tularensis*, whole-genome-sequencing, Germany, genotyping

## Abstract

*Francisella tularensis* is the causative agent of the zoonotic disease tularemia. In Germany, most human infections are caused by contact with infected hares. The aim of this study was to characterize *Francisella tularensis* subsp. *holarctica* strains isolated from hares in Germany and to develop bioinformatics tools to analyze their genetic relatedness. In total, 257 German isolates—obtained mainly from hares (*n* = 233), other vertebrate animals, and ticks, but also from humans (*n* = 3)—were analyzed within this study. Publically available sequence data from 49 isolates were used to put our isolates into an epidemiological context and to compare isolates from natural foci and humans. Whole-genome sequences were analyzed using core-genome Multi-Locus-Sequence-Typing, canonical Single Nucleotide Polymorphism (SNP) typing and whole-genome SNP typing. An overall conformity of genotype clustering between the typing methods was found, albeit with a lower resolution for canonical single SNP typing. The subclade distribution, both on local and national levels, among strains from humans and hares was similar, suggesting circulation of the same genotypes both in animals and humans. Whilst close to identical isolates of the same subclade were found distributed over large areas, small geographical foci often harbored members of different subclades. In conclusion, although genomic high-resolution typing was shown to be robust, reproducible and allowed the identification of highly closely related strains, genetic profiling alone is not always conclusive for epidemiological linkage of *F. tularensis* strains.

## 1. Introduction

Tularemia is a zoonotic disease that occurs mainly in the northern hemisphere and is caused by *Franciella (F.) tularensis*. The clinical signs and symptoms of the disease vary depending on the route of transmission and may present as ulceroglandular, oculoglandular, oropharyngeal or pneumonic tularemia [1,2]. The Centers for Disease Control and Prevention (CDC) consider this bacterium as a potential biological agent of category A, as the infection may have a major impact on an exposed human population [3].

In Germany, only the subspecies *F. tularensis holarctica* occurs in natural foci and humans usually acquire the disease due to contact with infected hares (*Lepus europaeus*) and less frequently from ticks, mosquitoes, contaminated dust, food, or water [4,5,6,7]. Two decades ago, most people considered tularemia to be extinct in Germany. The large natural outbreaks during and shortly after World War II were almost forgotten, and cases were reported only sporadically [8]. However, some serological studies indicated that *F. tularensis* could still be prevalent in the environment in Germany [9,10,11,12]. Wild boars (*Sus scrofa*) and foxes (*Vulpes vulpes*) proved to be useful bioindicators for the presence of the pathogen. They feed upon reservoir animals of *F. tularensis* such as small mammals and may be exposed to contaminated water, which can also be an important source of infection [11,13,14]. An outbreak among semi-free-living marmosets (*Callithrix jacchus*) in 2004 near Göttingen in Germany drew the attention to tularemia in Germany again [15]. In the following years, the awareness among veterinarians, physicians and hunters increased, and the diagnostic capabilities improved. In 2019, seventy-two human cases (SurvStat@RKI 2.0, https://survstat.rki.de; accessed 2020/09/11) and 207 cases among animals were reported in Germany (TSN online; accessed 2020/05/13).

In the last decade, isolates from many regions in Germany were collected and more recently, whole-genome sequencing was applied to elucidate the phylogeography of these genetically very closely related bacteria and to investigate outbreaks [16,17,18,19]. Currently, genotyping can be done using the open-source canSNPer software (canSNPer; https://github.com/adrlar/CanSNPer) to determine and analyze canonical single-nucleotide polymorphisms (canSNPs) [20,21,22,23,24]. Alternatively, core-genome MLST (cgMLST) (e.g., Ridom Seqsphere [17]; Bionumerics) and core-genome SNP calling can be performed. Previous studies have shown that *F. tularensis* subsp. *holarctica* subclades can be assigned predominantly to clade B.6 (biovar I, Erythromycin sensitive) and B.12 (biovar II, Erythromycin resistant), and both clades also occur in Germany [12,25,26]. Clade B.6 occurs predominantly in the south-western part and spreads to France and even further to the Iberian peninsula (“Iberian clone”) [21]. Clade B.12 occurs mainly in the north-eastern parts of Germany, Scandinavia, and eastern parts of Europe [12,27,28,29]. Comprehensive reviews of the European phylogeography of *F. tularensis* have been published previously [2,23,30].

The overall objective of this study was to perform whole-genome sequencing for in-depth phylogeography of *F. tularensis* subsp. *holarctica* from animals in Germany and to investigate genomic relatedness between samples from animals and humans. Finally, a bioinformatics pipeline for genotyping of *F. tularensis* subsp. was established for outbreak analyses.

## 2. Materials and Methods

### 2.1. Isolates

This study investigated a collection of strains mainly isolated from hare carcasses that were initially tested for tularemia in the German federal state laboratories. These laboratories kindly provided samples and isolates to the National Reference Laboratory of Tularemia at the Friedrich-Loeffler-Institut (FLI), Jena, Germany to confirm the diagnosis. A total of 270 *F. tularensis* subsp. *holarctica* German isolates obtained from humans, vertebrate animals, and arthropods were investigated. The metadata of all strains (i.e., year of isolation, district and federal state as geographical origin, and host) were provided in the Supplementary Data Files (Table S1).

The isolates were collected in the years 2006–2019 at the National Reference Laboratory of Tularemia, and some data had already been published in previous studies [12,19,31,32,33,34,35,36] Sequences of 143 samples have not been analyzed before (Table S1). All strains were characterized using a combination of independent methods including MALDI-TOF MS and conventional PCR assays as previously described [24,31]. The *F. tularensis* subsp. *holarctica* strains used in the present study were cultivated on cysteine heart agar (CHA, Becton Dickinson, BD Heidelberg, Germany) at 37 °C with 5% CO_2_ for 48 h from human specimens, animal samples, or ticks harvested from these carcasses. Before further handling isolates were inactivated at 95 °C for 20 min.

### 2.2. Whole-Genome Sequencing and Bioinformatics

Bacterial cells were harvested after 72 h of incubation at 37 °C with 5% CO2 and the DNA was extracted using QIAGEN Genomic-tip 20/G and a QIAGEN Genomic DNA buffer set kit (Qiagen, Hilden, Germany) according to the recommendations of the manufacturer. The DNA quality was examined by using a Qubit 2.0 fluorometer (Life Technologies, Darmstadt, Germany) with the Qubit dsDNA BR assay kit (Invitrogen, Carlsbad, CA, USA) and by agarose gel electrophoresis.

Paired-end Illumina (Illumina Inc., San Diego, CA, USA) sequencing of 66 isolates was performed at GATC (Konstanz, Germany) and for 190 isolates at IBIZ (Jena). In both cases, sequencing libraries were created using the Nextera XT DNA Library Preparation Kit (Illumina Inc., San Diego, CA, USA).

To compare the strains collected at FLI with other German strains, paired-end Illumina sequences of 45 previously published strains [18,26,37], including 14 strains previously published by this group were downloaded [18]. Additionally, one metagenomics sample of Illumina paired-end sequencing from an outbreak caused by contaminated must was included [16]. For the comparison with international reference strains, the strains LVS (SRA accession SRR2895624), FCS162 (SRR507783), FSC200 (GeneBank accession ASM16877v2), and OSU18 (ASM16877v2) were used.

Data analysis of paired-end data was performed with the pipeline WGSBAC [38,39] (v2.0.0). Raw sequencing data quality was controlled by WGSBAC with FastQC (v. 0.11.5) [40]. Coverage is a quality measure giving information on how often, in theory, each base of the genome was sequenced. Raw coverage was calculated as the number of reads multiplied with their average read length and divided by the genome size. Assembly of raw reads was performed with Shovill (v. 1.0.4) which is based on the SPAdes (v3.14.0) genome assembler [41]. The quality of assemblies was checked with QUAST (v. 5.0.2) [42]. N50 is a measure of the quality of assembled genomes which consist of contigs of different lengths [43]. N50 measures the continuity of the assemblies and is defined as the minimum length of all contigs that are required to cover 50% of the genome. This means that half of the genome sequence is in contigs that are larger than or equal to the size of N50 contig.

To identify contamination, Kraken 2 (v. 2.0.7 beta) [44] was used together with the database MiniKraken (v2) to classify reads and assemblies.

For genotyping, three approaches were used. (1) Genotyping utilizing pre-defined canonical SNPs using CanSNPer [24] based on assemblies. (2) Mapping based SNP typing using OSU18 as reference. Core genome SNPs were defined with Snippy (v. 4.3.6) [45] in standard settings. The pairwise SNP distance between isolates was calculated using the tool snps-dists (v 0.63). Phylogenetic trees based on core-genome SNPs were calculated using RaxML (v. 8.2.12) with the GTRGAMMA model [46]. Hierarchical clusters based on the number of core-genome SNPs were calculated using the function hclust of the statistical language R (v. 3.5.1). (3) To infer phylogeny based on cgMLST, the external software Ridom Seqsphere+ (v. 5.1.0) [47] was used in default settings with the specific core-genome scheme (cgMLST v2) for *Francisella tularensis* [48]. Ridom Seqsphere+ (v. 5.1.0) was also used to construct Minimum Spanning Trees (MSTs) and to perform clustering based on MSTs.

The raw sequencing data of the metagenomics data set from contaminated must were included in core-genome SNP analysis, while the assembled data of this sample were used for CanSNPs and cgMLST [16].

Simpson’s index of diversity is typically used to describe the discriminatory power of a typing method [49], denoting the probability that two randomly selected individuals from one sample will be classified into two different types. The web-based tool Comparing Partions was used to calculate Simpson’s index of diversity using the agreement measures Adjusted Rand and Adjusted Wallace [50]. To test the repeatability of sequencing and bioinformatics data analyses, one sample (08T0013) was sequenced in three independent runs. For interlaboratory comparison, DNA of six selected samples from FLI (Jena) was sent to the LGL (Oberschleißheim, Germany) for sequencing. The six strains belonged to the major clades B6. and B.12. WGS-data of all isolates were provided to the colleagues at FOI for verification of our analyses. All three partners have applied the pipeline WGSBAC.

## 3. Results

### 3.1. Strain Collection

Tularemia in hares (*Lepus europaeus*) is a reportable disease in Germany, so the vast majority of isolates at FLI was obtained from these animals. In total, sequence data of 305 isolates were analyzed including 270 isolates from this study, 45 German isolates from previous publications, and four reference strains (Table S1) [17,18,25]. The 270 isolates sequenced in this study were collected from vertebrate animals, humans and ticks: *Lepus europaeus* (*n* = 233), Gliridae (*n* = 2), *Meles meles* (*n* = 1), *Nyctereutes procyonoides* (*n* = 1), Simiiformes (*n* = 1), *Sus scrofa* (*n* = 2), *Vulpes vulpes* (*n* = 2), and humans (*n* = 3). Eleven isolates were collected from ticks (*Ixodes ricinus)* parasitizing on hares (*Lepus europaeus)*.

The 45 previously published isolates were collected from humans (*n* = 27), *Lepus europaeus* (*n* = 14), Castor (*n* = 2), *Sus scrofa* (*n* = 1), *Vulpes* species (*n* = 1) and *Nyctereutes procyonoides* (*n* = 1). One metagenomics sample from an uncommon outbreak potentially caused by contaminated grape must was included [16].

### 3.2. Whole Genome Sequencing Data

Genome sequencing of 270 isolates analyzed in this study yielded an average of 331,254 reads per isolate (range 196,954–24,323,202, Table S1). The mean coverage of the 270 isolates was 338 fold (range from 26 fold to 1594 fold). To check for putative contamination, the software Kraken2 was used which classifies each read (or contig) [41]. On the species level, the top hit for all 270 isolates was always “*Francisella tularensis*”. On average, 95% of the reads were classified as “*Francisella tularensis*”.

Genome assembly yielded an average genome size of 1,787,586 bp with a minimum of 1,760,711 bp (Table S1). The GC content was on average 32% with very little variation. The mean N50 of the 270 assembled genomes was 26.040 bp (range 13,573 bp–29,624 bp). Raw sequencing data of 270 isolates is available at the BioProject PRJEB40963 (Table S1).

To assess if sequencing and bioinformatics analysis were reliable and useful for outbreak investigations, one sample (08T0013) was tested in three independent runs at FLI (Jena). No SNP difference in the core-genome was detected comparing the three sequencing data sets. Moreover, a subset of six DNAs of strains representing the two major clades were shared with the laboratory in LGL (Oberschleißheim, Germany) and sequenced and analyzed in both laboratories. The isolates could be assigned to the correct subclades and the results in both laboratories did not differ even by a single SNP in any of the six specimens (Figure S1).

### 3.3. Genome Structure of Francisella tularensis in Germany

To get a broad picture of the relatedness of the isolates among each other, the well-established canonical SNP typing [24] was first used. For all 305 analysed isolates, canonical SNP typing was successfully performed. The German isolates belong to major clade B6 (*n* = 199) and B.12 (*n* = 102) (Table S1).

**Figure 1:**
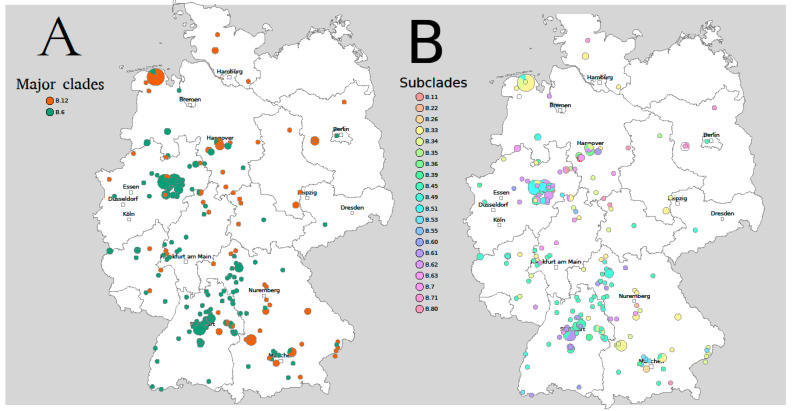
CanSNP-Typing of *Francisella tularensis* in Germany. (**A**) Major clades, (**B**) Subclades. The diameter of the circles corresponds with the number of *Francisella tularensis* subsp. *holarctica* isolates in this region. The subclades assigned based on canSNP typing occur in different regions, but also overlap in large areas. Figure adapted from RidomSeqSphere+.

The major clades B.6 and B.12 are overlapping in Germany, but B.6 is more dominant in the South-West, while B.12 occurs predominantly in the East (Figure 1A).

German strains belong to 25 different subclades: B.45 (*n* = 74) is the most prevalent subclade followed by B.33 (*n* = 41), B.34 (*n* = 26), and B.49 (*n* = 25). The subclades assigned based on canSNP typing occur in different regions but overlap in large areas (Figure 1B).

To investigate the genome structure of *F. tularensis* isolates in Germany in more detail, cgMLST and core-genome SNP typing was performed. Using cgMLST, the genome sequences of the isolates are mapped onto 1147 pre-defined cgMLST targets [48]. For 305 samples, on average, 99.1% of the targets were classified as “Good Targets” by the Ridom SeqSphere (range 95–99.7%, Table S1). For core-genome SNP. The strain FSC200 was ignored as no read data were available. The remaining 304 strains contributed to a core-genome with 1536 SNPs. The pairwise distance between two isolates is, on average, 226.6 SNPs and ranges from 0 to 490 SNPs (Table S3). In general, both core-genome methods were in accordance with the results obtained with canonical SNP typing and reproduce major clades and subclades (Figures S2 and S3). CgMLST resulted in clades corresponding to the clades B.6 and B.12 when a cut-off of 200 different alleles was applied. However, both core-genome methods allow for higher resolution as indicated by a Simpson’s index of diversity for cgMLST of 0.979 and core-genome SNPs of 0.991 than canonical SNP typing (Table S2). For example, the 188 isolates in the canonical subclade B.45 had an average pairwise distance of 59.2 SNPs (range 0–487) (Table S3).

As this study concerns the genomic structure of *F. tularensis* from hares in Germany, clustering based on cgMLST and core-genome SNPs to identify clonal outbreaks was perfomed. To this end, the cut-offs 1, 5, and 10 were tested for the number of alleles (cgMLST) or the number of core-genome SNPs differences, respectively (Table S1, Figures S2 and S3). Both methods provided similar results. Comparing clustering based on one allele differences in Minimum Spanning Trees (MST) of cgMLST to clustering based on maximal one core-genome SNP, we saw mainly concordance, while sometimes MST based clustering missed some isolates. In conclusion, we analyzed clustering based on 1 SNP distance in more detail, as it provided the highest resolution.

**Figure 2:**
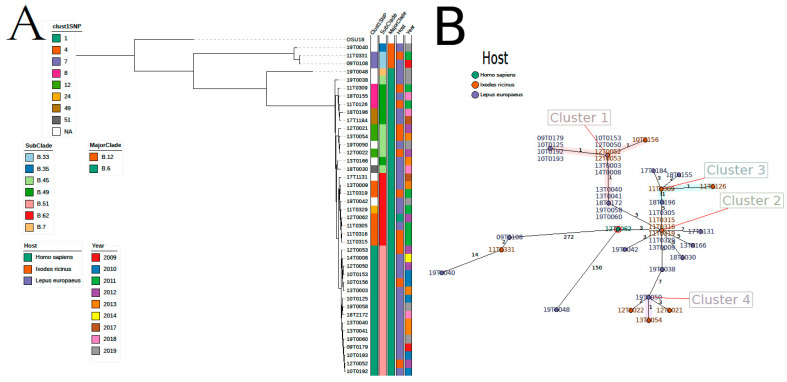
Epidemiological situation in the region of Soest based on core-genome single-nucleotide polymorphisms (SNPs) (**A**) using clustering of whole Germany (Figure S2) and based on cgMLST (**B**). In Soest highly similar isolates from hares and humans exist. However, the region also contains highly diverse isolates. Core-genome SNPs were defined using Snippy and the tree was calculated by RAxML. RidomSeqSphere+ was used to calculate cgMLST and to create the Minimum Spanning Tree.

This clustering grouped 189 isolates into 55 clusters (Table S1, Figure S3). The three largest clusters (cluster 1–3) contained 18, 16, and twelve isolates, respectively. Thirty-five clusters (mainly among hares) consisted of only two isolates. Most of the large clusters were found in one specific region (Table S1, Figure S4). Bacteria cluster 1 was mainly isolated in North Rhine-Westphalia, and bacteria from cluster 3 from Baden-Wuerttemberg. On the other hand, cluster 2 spread over different regions, primarily in central and southern Germany. The largest cluster (cluster 1) based on 1 SNP distance contained 18 isolates (Table S1, Figure S3) and was in complete concordance to the cluster based on 1 allele difference using cgMLST. Sixteen of these 18 isolates were from the county Soest belonging to the federal country North Rhine-Westphalia. As we found another cluster based on 1 SNP difference containing six isolates from Soest, we decided to analyze this region in more detail. Forty-one isolates were collected and sequenced in the county of Soest. While the majority of isolates were assigned to major canonical SNP clade B.6, three strains belonged to clade B.12 (Figure 2, Table S1). Seven canonical subclades exist in the county of Soest, where B.51 (*n* = 16) is the most abundant followed by B.7 (*n* = 7) and B.45 (*n* = 6) and B.49 (*n* = 6). Regarding core-genome SNPs, the average pairwise SNP distance is 78.5 (range 0–463) (Table S3). Of the 55 clusters based on maximal 1 core-genome SNP distance, bacteria isolated in the region of Soest were contained in eight clusters (Figure 2A, Table S1), which were also supported by cgMLST (Figure 2B). The largest cluster (cluster 1) spanned over one decade (2009–2019) and contained 13 isolates collected from hares and three from ticks *(Ixodes ricinus).* The second largest cluster (cluster 4) contained five isolates from 2011–2013 collected from two hares, three ticks *(Ixodes ricinus)*, and one human case. This cluster was supported by cgMLST and contained five more isolates outside Soest. Three isolates belonged to cluster 8 and two to cluster 7. Both clusters contained further isolates from the region of Wittmund (Lower Saxony).

### 3.4. Genotyping Connection between Isolates from Animals and Humans

To assess the relatedness of *F. tularensis holarctica* from hares and humans, the genomes of isolates from animals (mainly hares) and human patients were compared. On the level of major canonical SNP clades, 75% of human isolates were assigned to clade B.6 compared to 65% isolates from hares (Table S4). Isolates from hares and humans shared thirteen canonical subclades being B.45 the most abundant subclade for both genera. While B.52 was isolated only from one human individual, eight subclades (B.80, B.11, B.26, B.35, B.55, B.60, B.22, B.24) were unique for hares.

To identify potential clonal connections, clusters of strains that had a maximum of 1 SNP difference were identified. One cluster (cluster 2) consisting of isolates obtained from five humans and nine hares was identified. These bacteria were isolated between 2010 and 2019 in central and southern Germany (Table S1, Figure S3). Isolates of this cluster also grouped together when analyzed using cgMLST. As mentioned above, cluster 4 contained bacteria from the region of Soest isolated from hare ticks and one human. Cluster 10 contained one human isolate from 2016 in Baden-Wuerttemberg and two bacteria isolated from hares in 2009 in the county of Wittmund (Lower Saxony). Cluster 11 contained two human isolates from Lower-Saxony (2015) and one isolate from a hare isolated 2011 in Gießen (Hesse) and was supported by clustering based on cgMLST with one allele as the cut-off. Cluster 19 contains bacterial isolates from two hares and one human isolated between 2015–2016 in Mecklenburg-Vorpommern and was also supported by cgMLST. Cluster 39 contained isolates from a human patient (2017, Rhineland-Palatinate) and a non-human primate (Simiiformes; 2015, Berlin).

## 4. Discussion

Epidemiological tracking of *F. tularensis* is challenging because extensive phylogenetic diversity can be found within single counties in Germany. At the same time, almost identical isolates can spread over large distances and long periods. Therefore, it is difficult to link specific genetic types with specific geographic locations and to determine the timeframe of the spread of these bacteria [24,51,52]. Hence, genetic profiling data alone is not sufficient to establish an epidemiological link without taking into account time, place, and local hosts. It is not fully understood why genetically almost identical isolates can spread over large distances. However, migratory birds may play a role as ticks collected from them can carry *F. tularensis* [53].

The genetic diversity of *F. tularensis* in Germany was investigated in the past, but until now, the number of tested strains and published whole-genome sequences were relatively low [12,26]. Since more *F. tularensis* strains had been collected, an update on the situation in Germany was needed. This was of urgent interest, as the number of cases in humans and animals has multiplied since the beginning of our studies at the national reference laboratory in 2009. It has to be underlined that the federal laboratories are not obliged to send isolates of *F. tularensis* to the national reference laboratory. So the collection of strains was only possible because of the excellent work of these colleagues and their will to cooperate and to facilitate our scientific work. Based on these joint efforts, a representative collection of isolates and publicly available genomes of *F. tularensis* isolates from Germany was analyzed in this study.

As previously shown, biovar I and II are prevalent in Germany. Identical canSNP clades occur in different federal states, and an overlap of clades was observed even on the county level. In most counties, up to two clades were identified, but in the county Soest (85.81 km^2^, NRW), strains of five different clades (Table S1) were found. In Sweden, single outbreaks of tularemia have been shown to be caused by several clones of *F. tularensis* in the past.

The major clades B.6 and B.12 are overlapping in Germany, but a predominance of major clade B.6 in the south-west was found, while B.12 dominates in the East. The reason for this geographical segregation is unclear, but an ecological barrier may have been active in the past or that segregation simply occurred by stochastic processes. In addition, we also found coexistence of multiple clones in small geographical foci in Germany, a finding previously reported from local outbreaks elsewhere [23,54,55,56,57].

Strains belonging to the major clade B.6 were mainly isolated in the (south-)western part of Germany. Subclade B.7 is known to occur in Scandinavia (Norway, Sweden) and was also found in three federal states in Germany (Table S1). While five subclades exist only once in Germany, B.45 was the most frequent subclade. Subclade B.11 was found in Germany, but also in The Netherlands and France [58,59].

The major clade B.12 was found to be present in Germany with several subclades.

The individual subclade B.22 has previously only been found in Sweden and was thought to be geographically confined, but we isolated such a strain also from a hare in Germany in 2015 (15T0794 in the village Wendelstein, county Roth, Bavaria) [51]. Based on cgMLST, the nearest neighbor was the Swedish reference strain FSC200, which differed in only three alleles. We could not determine the difference regarding the number of SNPs, because only the assembly and no reads of FSC200 were available [60].

The subclades B.26, B.33, B.34, B.35, and B.36 are on the same phylogenetic branch and, therefore, presumably represent the timeline of evolution. The most ancestral subclade B.26 was found in Bavaria close to Munich (two isolates). The descendant (derived) subclades B.33 and B.34 spread all over Germany. Subclade B.35 was found exclusively in the northern part of Germany, and subclade B.36 was found only around the city of Hannover. This might indicate that the older subclades had sufficient time to spread over a large area, whereas the youngest descendants occurred only in a relatively small area. Members of this phylogenetic branch have previously been found in Germany, Hungary, The Netherlands, Bulgaria, Austria, Switzerland, and Scandinavia [16,29,58,61]. Subclade B.39 was found, e.g., in France [59], Sweden [22], and Finland [62], but four strains from hares (this study) and one from humans were also isolated in Germany.

Subclade B.71 belongs to the major clade B.12, but looking at core-genome SNPs (Figure S3) and cgMLST (Figure S4), this subclade is quite distant to other B.12 strains and seems to be in the middle of B.6 and B.12.

The phylogeographic pattern of tularemia was shown to be complex (e.g., in Sweden and France) and genotyping based on canSNPs also has some limitations regarding the resolution [51,59]. In our study, this was especially true for the major clade B.6. New canSNPs can be defined while cgMLST and core-genome SNP typing help to further differentiate these strains. The largest subclade of German B.6 strains is the subclade B.45. However, subclade B.45 can be differentiated into more detailed genomic clusters using cgMLST and core-genome SNP typing. Such a high phylogenetic resolution may help to understand the infection routes of human cases. Genotypically similar (or even identical) isolates may be epidemiologically linked in a recent event but do not necessarily have to be. For example, cluster 2 with highly similar genomes (max. 1 SNP difference) isolated in a large region covering central and southern Germany was identified. Here, even a higher resolution would be needed. This might be achieved by combining long reads with short reads and thus generating complete hybrid assemblies. Within complete hybrid assemblies, whole-genome SNP analysis instead of core-genome SNP analysis is possible. The previously described affinity propagation method [36] for clustering is in accordance to major canonical clades and subclades, but does not provide sufficient discrimination for outbreak analysis.

A limitation of the study was the overrepresentation of isolates from some regions of Germany due to the low number of hares and reported cases in the eastern parts of Germany. Since 2003/4, the number of hares has decreased continuously in Germany (568,548 hunted animals in 2003/4; 184,666 in 2017/2018; source: “Wildtier-Informationssystem der Länder Deutschlands—Jahresbericht 2018”; p.6-12). The decrease in animals can be explained by unfavorable climate changes, intense agriculture, and a higher number of predators (e.g., foxes, raccoon dogs, raccoons).

## 5. Conclusions

We found that there is no clear geographic relationship between the spread of biovar I and II of *F. tularensis* subsp. *holarctica* in Germany and that up to five different subclades can be found in a single county. Whole-genome sequencing typing is indispensable to investigate outbreaks caused by monomorphic pathogens such as *F. tularensis*. We recommend using whole-genome SNP typing or cgMLST in local outbreak investigation as they have a very high resolution and canSNP analysis for surveillance studies on a larger region or national level and the assignment to subclades is in line with the global nomenclature. Furthermore, we could show that SNP typing delivered robust and consistent inter-laboratory typing results. This study provides a dataset that will be used to provide detailed epidemiological reports to the federal laboratories for outbreak analyses and should provide a comprehensive overview of the current situation in Germany.

## Supplementary Materials

The following are available online at https://www.mdpi.com/2076-2607/8/12/1932/s1, Table S1: Metadata of collected isolates and sequencing results including quality measures, canonical SNP types and clustering; Table S2: Simpson’s index of diversity and confidence intervals (CI) for the applied typing methods; Table S3: Pairwise SNP distance of 304 isolates; Table S4: Number of major clades (tab 1) and sub clades (tab 2) per host. Figure S1. Six isolates were sequenced at FLI and the LGL (Oberschleißheim, Bavaria). Subsequently, core genome SNP analysis was performed in both laboratories. No differences were found which indicates that sequencing and the bioinformatics pipeline were robust and the results were reproducible; Figure S2: Phylogenetic tree (RaXML) based on core-genome SNP alignment including metadata and clustering information. Figure S3: Minimum Spanning Trees from cgMLST analysis. (**A**) Clustering using 200 alleles differences is in accordance to major canonical SNPs. (**B**) Subclades mainly follow the MST structure. Figure S4: Geographical map of clustered isolates based on maximal 1 SNP distance. Only clusters with at least four isolates are visualized (Figure S2, Table S1).
