# Supplementary figures and images for: Genotyping of Francisella tularensis subsp. holarctica from Hares in Germany
Source: Microorganisms. 2020 Dec 5;8(12):1932. doi: 10.3390/microorganisms8121932 (PMC7761992; doi:10.3390/microorganisms8121932)

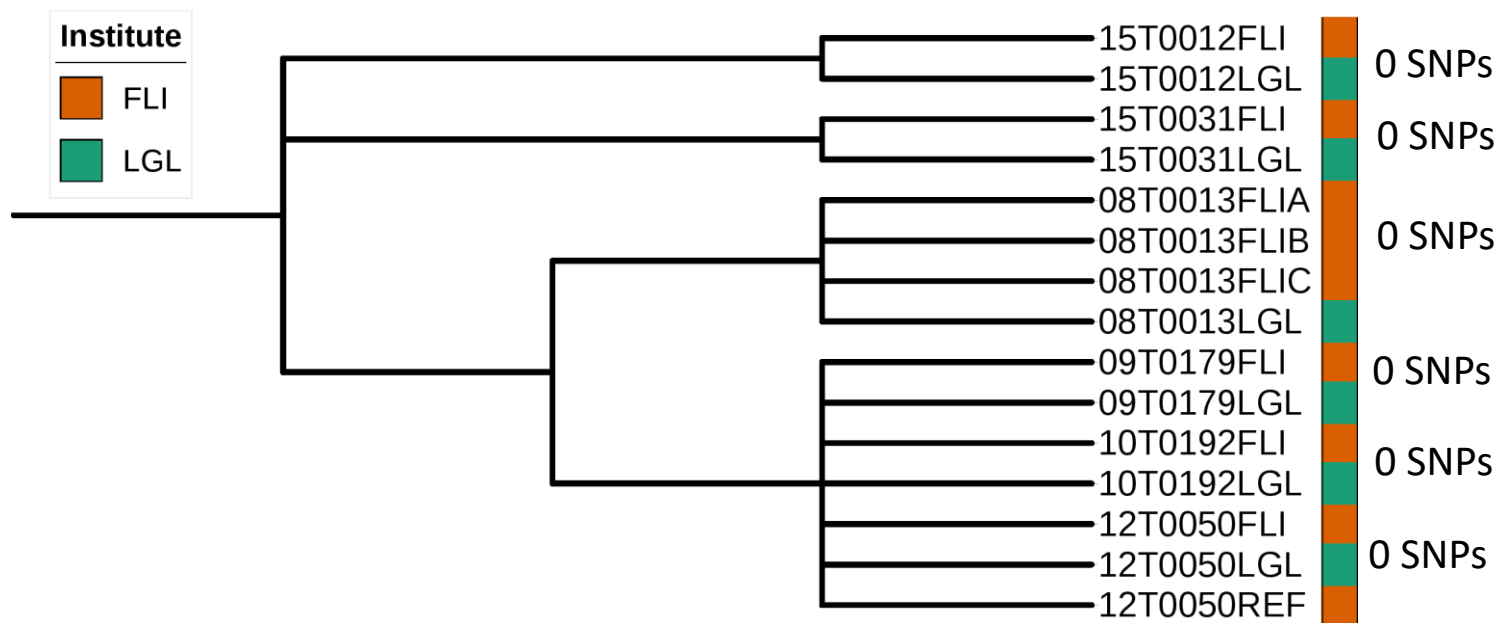

Supplement: Supplementary file 1 [file microorganisms-08-01932-s001.zip › Supplement/FigureS1.pdf]

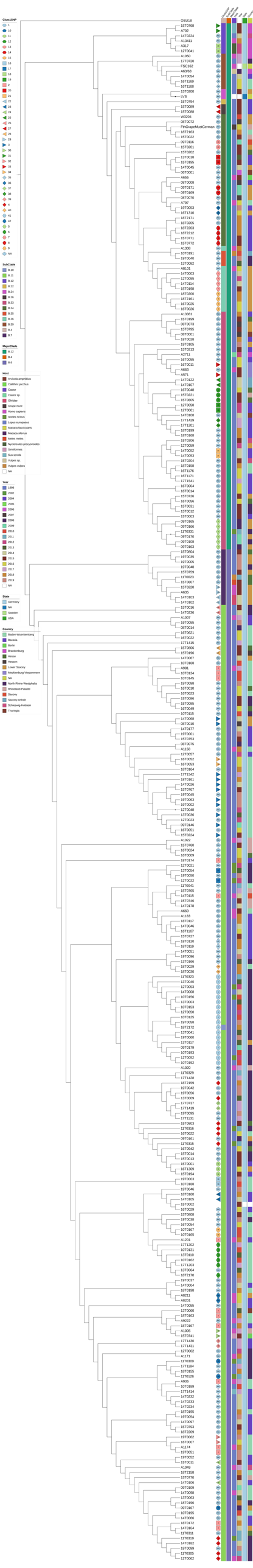

Supplement: Supplementary file 1 [file microorganisms-08-01932-s001.zip › Supplement/FigureS2.pdf]

# A

## Major clades

- B.4
- B.12
- B.6

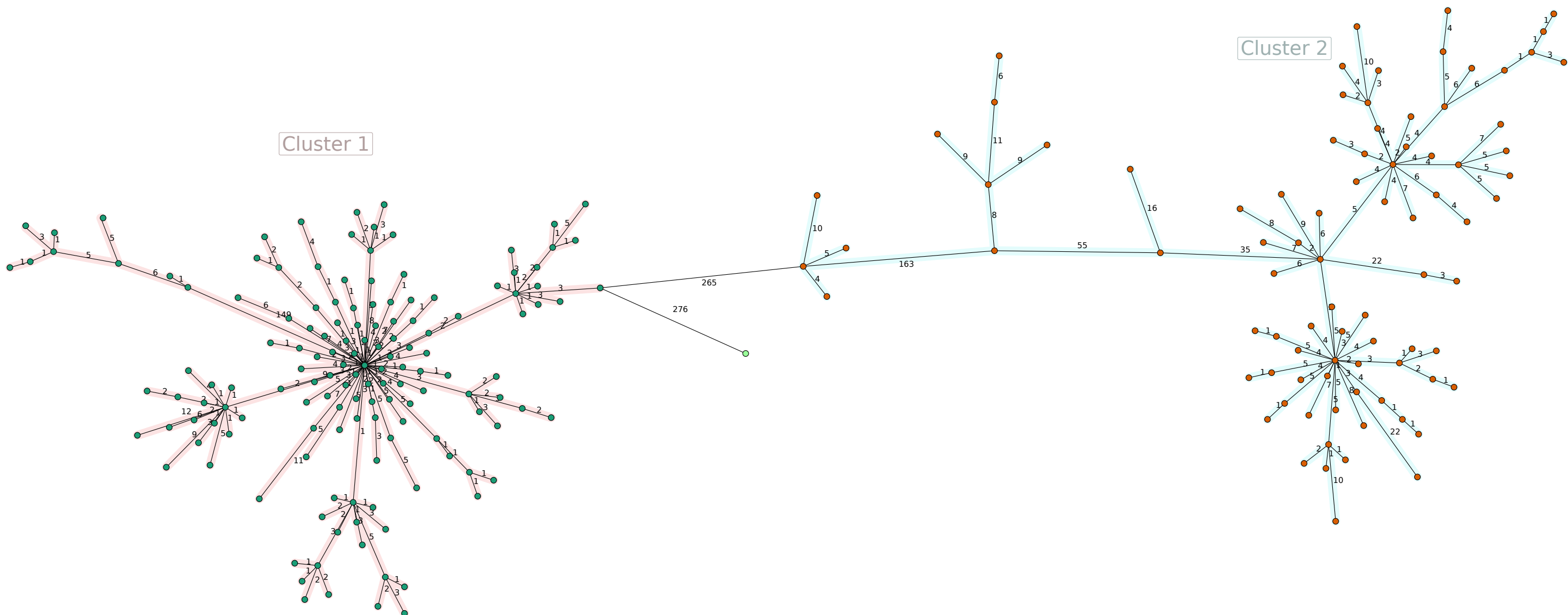

# B

## Subclades

- B.11
- B.22
- B.26
- B.33
- B.34
- B.35
- B.36
- B.39
- B.45
- B.49
- B.51
- B.53
- B.55
- B.60
- B.61
- B.62
- B.63
- B.7
- B.71
- B.80

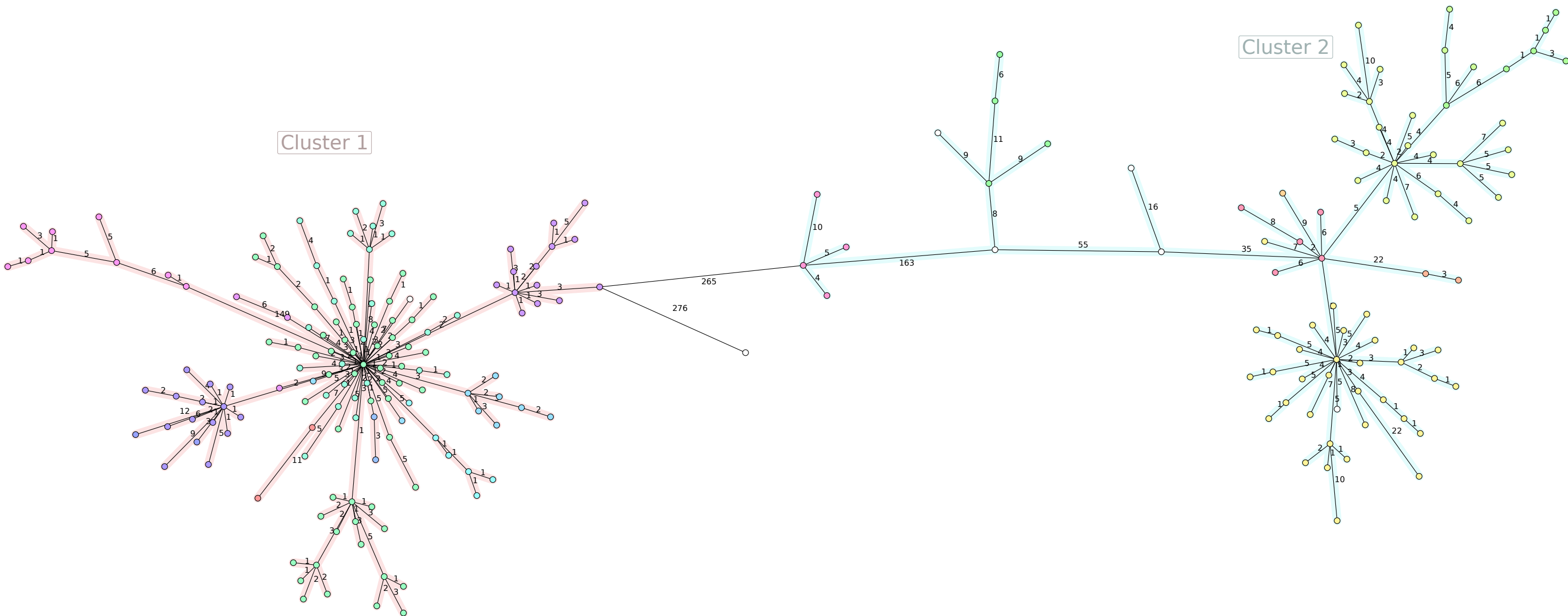

Supplement: Supplementary file 1 [file microorganisms-08-01932-s001.zip › Supplement/FigureS3.pdf]

# Clust1SNP

- 1
- 2
- 3
- 4
- 5
- 6
- 7
- 8
- 9

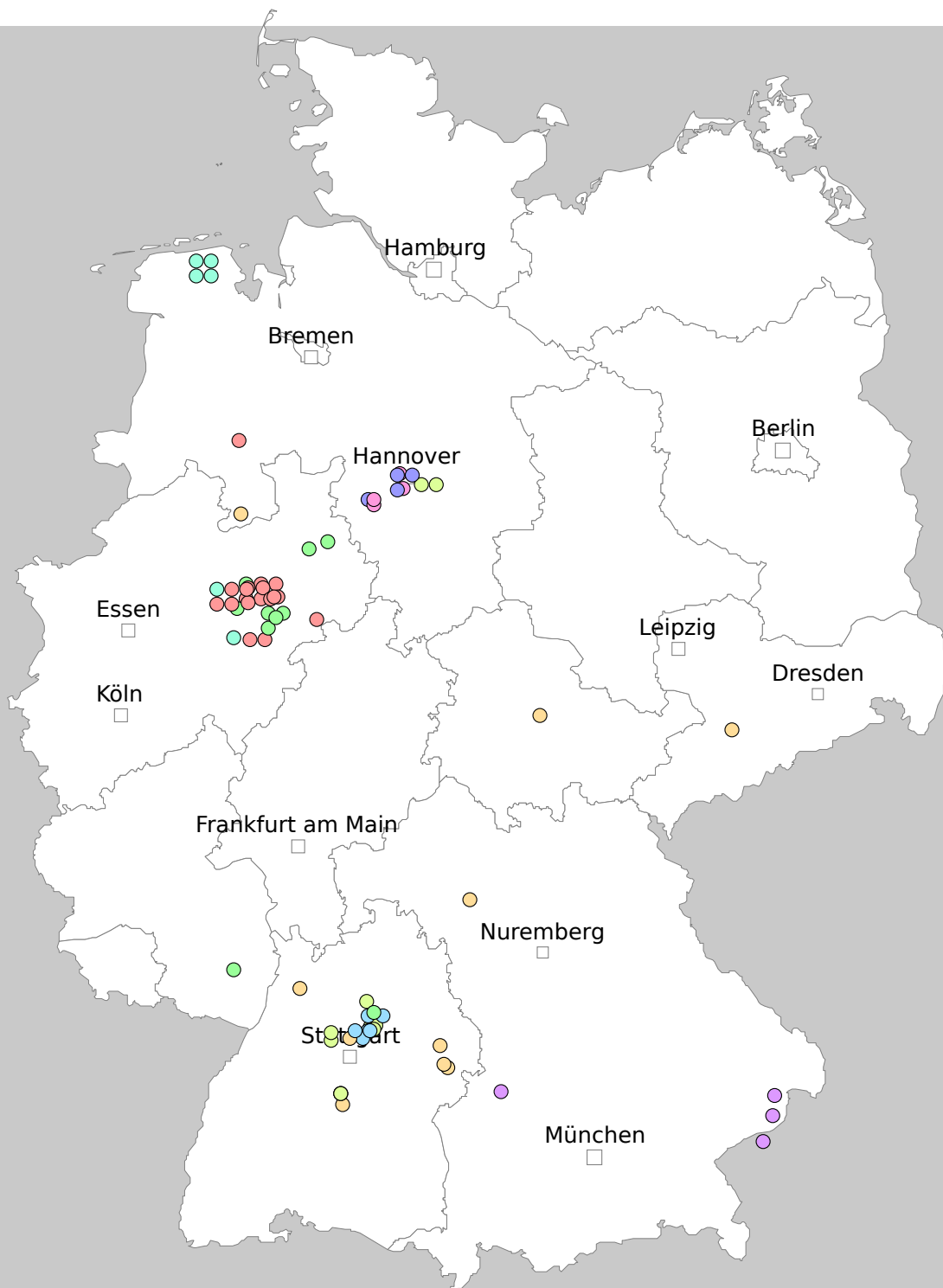

Supplement: Supplementary file 1 [file microorganisms-08-01932-s001.zip › Supplement/FigureS4.pdf]
